# Comparison of Leaf Sheath Transcriptome Profiles with Physiological Traits of Bread Wheat Cultivars under Salinity Stress

**DOI:** 10.1371/journal.pone.0133322

**Published:** 2015-08-05

**Authors:** Fuminori Takahashi, Joanne Tilbrook, Christine Trittermann, Bettina Berger, Stuart J. Roy, Motoaki Seki, Kazuo Shinozaki, Mark Tester

**Affiliations:** 1 Biomass Research Platform Team, RIKEN Center for Sustainable Resource Science, Koyadai, Tsukuba, Ibaraki, Japan; 2 Gene Discovery Research Group, RIKEN Center for Sustainable Resource Science, Koyadai, Tsukuba, Ibaraki, Japan; 3 Australian Centre for Plant Functional Genomics, School of Agriculture, Food & Wine, University of Adelaide, Glen Osmond, Australia; 4 The Plant Accelerator, Australian Plant Phenomics Facility, School of Agriculture, Food & Wine, University of Adelaide, Glen Osmond, Australia; 5 Plant Genomic Network Research Team, RIKEN Center for Sustainable Resource Science, Suehiro-cho, Tsurumi-ku, Yokohama, Kanagawa, Japan; Huazhong University of Science & Technology(HUST), CHINA

## Abstract

Salinity stress has significant negative effects on plant biomass production and crop yield. Salinity tolerance is controlled by complex systems of gene expression and ion transport. The relationship between specific features of mild salinity stress adaptation and gene expression was analyzed using four commercial varieties of bread wheat (*Triticum aestivum*) that have different levels of salinity tolerance. The high-throughput phenotyping system in The Plant Accelerator at the Australian Plant Phenomics Facility revealed variation in shoot relative growth rate and salinity tolerance among the four cultivars. Comparative analysis of gene expression in the leaf sheaths identified genes whose functions are potentially linked to shoot biomass development and salinity tolerance. Early responses to mild salinity stress through changes in gene expression have an influence on the acquisition of stress tolerance and improvement in biomass accumulation during the early “osmotic” phase of salinity stress. In addition, results revealed transcript profiles for the wheat cultivars that were different from those of usual stress-inducible genes, but were related to those of plant growth. These findings suggest that, in the process of breeding, selection of specific traits with various salinity stress-inducible genes in commercial bread wheat has led to adaptation to mild salinity conditions.

## Introduction

Bread wheat (*Triticum aestivum*) is now grown on a greater area than any other cereal crop, but is only moderately tolerant to salinity stress [1]. With soil salinity affecting one-fifth of irrigated agricultural land worldwide [2], there is an urgent need to improve the salinity tolerance of bread wheat to meet future global food requirements.

Shoot Na^+^ exclusion is only one component of plant salinity tolerance, and some studies have found no clear correlation between plant salinity tolerance and Na^+^ exclusion under conditions of moderate salinity stress [3–6]. Another important, but not well characterized, tolerance trait is the shoot ion accumulation independent tolerance mechanism (also called “osmotic tolerance”), which is the ability of plants to maintain growth rates after initial exposure to salinity stress that is independent of the extent of shoot ion accumulation [1, 7]. This osmotic tolerance mechanism is important for maintaining tissue expansion and tillering during the early stages of salinity stress before Na^+^ and Cl^-^ ions accumulate to toxic concentrations in the shoot. Plants with poor osmotic tolerance will show significant reduction in growth immediately after salt application. This reduction in growth will continue throughout the time of exposure to salinity, resulting in plants with reduced biomass and yield [1, 5, 7]. Unfortunately, the mechanisms behind osmotic tolerance are poorly understood, and no gene for increased tolerance has yet been linked specifically to this trait. To advance breeding for salinity tolerance in bread wheat, it is imperative to identify the genes important for osmotic stress tolerance.

It is well known that thousands of genes are differentially regulated in response to a range of abiotic stresses including salinity and drought [8, 9], due in part to plant stress-tolerance mechanisms in the early phase of stress responses. In wheat, several genes have been reported to be induced rapidly after salinity stress. The salinity stress-inducible transcription factor (TF), *TaWRKY10*, has been found to be important in regulating drought responses and the accumulation of reactive oxygen species [10]. Wheat *calcineurin B-like protein-interacting protein kinase 29* (*TaCIPK29*) and *sucrose nonfermenting 1-related protein kinase 2* (*TaSnRK2*) are also induced by abiotic stress and are important in plant stress signaling pathways that allow rapid control of multiple tolerance mechanisms [11–18]. However, it is still unclear whether these salinity stress-inducible genes are associated with salt or osmotic stress tolerance.

In the present study, four commercial bread wheat cultivars were exposed to salinity stress conditions to characterize the transcriptional response to early stages of osmotic stress in the key tissue affecting growth over this time period, the leaf sheaths. The high-throughput phenotyping system of The Plant Accelerator at the Australian Plant Phenomics Facility in South Australia allowed the continuous measurement of growth rates to determine the osmotic stress tolerance component of all four cultivars. A comprehensive evaluation based on gene expression profiles of leaf sheaths and phenomics highlighted gene sets associated with individual physiological traits. We discuss the correlation between the salinity stress tolerance of these commercial salinity-tolerant cultivars and their leaf sheath transcriptomes during the early response of shoots to salinity stress.

## Materials and Methods

### Plant material and growth conditions

Four commercial varieties of bread wheat (*Triticum aestivum*) were used in this study–Berkut, Krichauff, Gladius and Drysdale. Gladius and Drysdale are parents of a mapping population developed in Adelaide, and Berkut and Krichauff are the parents of another mapping population. Krichauff (pedigree: Wariquam//Kloka/Pitic62/3/Warimek/Halberd/4/3Ag3Aroona), Gladius and Drysdale (pedigree: Hartog*3/Quarrion) are relatively recent commercial Australian cultivars; Berkut (pedigree: Irena/Babax//Pastor) was developed by the International Maize and Wheat Improvement Centre (CIMMYT) in Mexico. Gladius was derived from a series of crossings among derivatives of RAC875, Krichauff, Excalibur, and Kukri. No specific permissions were required for the cultivation in the greenhouse of The Plant Accelerator at the Australian Plant Phenomics Facility described in the text because the non-transformant crops were grown and analyzed in the hermetically-closed greenhouse approved for academic plant science. The field studied did not involve endangered or protected species.

Seeds were soaked in reverse osmosis (RO) water at room temperature for 2 h. The water was drained and the seeds placed in the dark at 4°C for 2 days in sealed 50 mL polypropylene tubes. Evenly sized imbibed seeds were selected for sowing and four seeds per pot were planted 2 cm deep into 145 cm diameter by 190 cm height free-draining pots. The soil substrate was a potting mix consisting of 50% (v/v) University of California mix, 35% (v/v) peat mix and 15% (v/v) clay loam, designed specifically for use on The Plant Accelerator’s conveyor system. Plants were watered to a gravimetric water content of 25% (w/w). The seedlings were grown in natural light in a temperature-controlled greenhouse, with a daytime temperature of 22°C and 15°C at night. The pots were randomly placed in the greenhouse. At the time of emergence of leaf 2, seedlings were thinned, leaving one seedling per pot. Two experiments were performed with different concentrations of NaCl to measure the effects of mild salinity stress. The first experiment measured the growth response of wheat seedlings treated with 0 and 75 mM NaCl. The second experiment measured the growth response of seedlings treated with 0 and 100 mM NaCl, with RNA also extracted from leaf sheath tissue for microarray analysis. The experiments were conducted consecutively during the winter months of 2011 (from late May until early August) in Adelaide, South Australia (34°58'17.00"S, 138°38'23.00"E).

### Assay for growth under salt stress conditions

Following emergence of the third leaf, the free-draining pots were placed in a deep saucer and loaded onto the conveyers of The Plant Accelerator’s automated imaging system [19], using a randomized block design (Brien *et al*, 2013), as shown in S1 and S2 Figs. Daily imaging was performed as described by Brien *et al*. (2013). Seedlings were watered to 25% (w/w) gravimetric water content. At the emergence of leaf four, the salinity treatment was applied as described by Berger *et al*. (2012). The salinity treatment solution (in a volume of 210 mL) was added to the saucer for uptake through the base of the pot, a process requiring approximately 2 h. After treatment, the soil water content was measured to be in the order of 35% (w/w).

For the first experiment, after application of the treatments, the pots contained 0 or approximately 50 mM NaCl which, over a period of 6 to 7 days was maintained at 0 or increased to 75 mM by evaporation and transpiration to reach the target gravimetric water content of 25% (w/w). For the second experiment, after treatment the soil contained 0 and approximately 70 mM NaCl and was maintained at 0 or increased to 100 mM NaCl over 6 to 7 days, as described above. Watering recommenced when the soil water content reached 25% (w/w) and NaCl in the soil solution was at the designated concentration. The method was designed to impose gradual salinity stress on the seedlings.

The LemnaTec Scanalyzer 3D (LemnaTec GmbH, Aachen, Germany) at The Plant Accelerator was used for daily image acquisition [20]. Two red-green-blue (RGB) images were captured from the side at a 90° rotation to each other and one RGB image from above. LemnaGrid software (LemnaTec GmbH, Aachen, Germany) was used for image processing to determine the projected shoot area—the sum of pixels identified as being part of the plant in each image [19, 21].

To measure shoot growth during the plant osmotic response period, the projected shoot area from days 0 to 8 after salinity application was recorded. At this stage, plants were still young and the use of an exponential growth model is still appropriate. There is also a linear correlation between projected shoot area and shoot biomass, for *Triticum monococcum* [22] and for bread wheat (B. Berger, unpublished results), so that projected shoot area can be used directly to measure relative growth rates (RGRs). The projected shoot area was plotted over time and exponential growth curves were fitted to determine RGR [19, 22]. The salinity tolerance index (STI) was determined:

$$\text{STI}=\frac{\left(\text{RGR under saline conditions}\right)}{\left(\text{RGR under control conditions}\right)}$$

Over the time course of this experiment, this approximates to a measure of the tolerance of plants to the osmotic phase of salinity toxicity, the shoot ion accumulation independent tolerance (Roy et al., 2014).

### Stress treatment and extraction of RNA for microarrays

Separate replicate pots of each cultivar were grown under conditions identical to those of the second experiment (0 and 100 mM NaCl) for destructive sampling of shoot material for RNA extraction at four separate time points. The leaf sheaths of seedlings of each cultivar were collected immediately before (day 0) or 1, 2, and 3 days after application of 0 or 100 mM NaCl. Samples from three replicate seedlings were taken at each time point under both control and salt stress conditions, and total RNA from the sheaths was isolated using Trizol-modified reagent [23], and then used for the preparation of Cyanin-3 (Cy3)-labelled cRNA probes. Dye incorporation and cRNA yield were checked with the NanoDrop ND-1000 Spectrometer. The Cy3-labelled cRNA was fragmented and hybridized to the Agilent wheat microarray (Design ID: 0222297) according to the manufacturer’s guidelines. All microarray data have been deposited in the ArrayExpress database (https://www.ebi.ac.uk/arrayexpress/; accession number E-MTAB-3270).

### Data mining of gene expression with Agilent wheat gene expression microarray

Microarray experiments were performed with the four bread wheat cultivars, Berkut, Krichauff, Gladius and Drysdale, using a 44K wheat gene expression array with a one-color gene expression microarray solution (Agilent Technologies). Each analysis was repeated in triplicate as biological replicates—a total of 84 microarray experiments were performed according to the manufacturer’s manual. Genes displaying a signal value of >1000 were selected for analysis. Feature extraction and image analysis software (GeneSpring GX version 12.5; Agilent Technologies) was used to locate and delineate each spot in the array and to perform a statistical analysis of the intensity of each spot using the Lowess method (*p* < 0.01). The statistical significance of gene expression was tested using the ANOVA analysis of variance test combined with a Benjamini and Hochberg false-discovery-rate multiple correction algorithm with a corrected *q* value of <0.01. The criterion for the fold change threshold selection was a greater than five-fold change of expression. The changes in gene expression were calculated as follows:

For expression analysis under control conditions:

$$\frac{\left(\text{expression at}\;1,2\;\text{or}\;3\;\text{days under control conditions in each cultivar}\right)}{\left(\text{expression at day}\;0\;\text{under control conditions in each cultivar}\right)}$$

Or, for expression analysis under salinity conditions:

$$\frac{\left(\text{expression at}\;1,2\;\text{or}\;3\;\text{days under salinity conditions in each cultivar}\right)}{\left(\text{expression at}\;1,2\;\text{or}\;3\;\text{days under control conditions in each cultivar}\right)}$$

To identify functional descriptions of genes, EST or probe sequence data were searched against the following nucleotide datasets using the EST data of the NCBI (http://www.ncbi.nlm.nih.gov/), the cDNA data of rice derived from RAP-DB v.2 (http://rapdb.dna.affrc.go.jp/) and the cDNA data present in TAIR release 10 (ftp://ftp.arabidopsis.org/home/tair/Sequences/blast_dataset/). The top-scoring hit for each query was selected for gene annotation with a threshold E-value of <1E-5, and the Load ID was used for the functional classification using the gene ontology (GO) of agriGO (http://bioinfo.cau.edu.cn/agriGO/), amiGO 2 (http://amigo.geneontology.org/amigo) and the Gene Ontology (http://www.geneontology.org/). The percentage of identified genes was calculated as follows:

$$\text{percentage}\;(\%)=\frac{\left(\text{number of the genes classified by the GO term}\right)}{\left(\text{total number of genes used for the classification}\right)}\times 100$$

Gene expression profile charts under stress conditions were searched against the following datasets using the eFP Browser (http://bbc.botany.utoronto.ca/efp/cgi-bin/efpWeb.cgi) for *Arabidopsis* and the RiceXPro (http://ricexpro.dna.affrc.go.jp/) for rice.

### Quantitative RT-PCR analysis

Five microgram of total RNA was used for cDNA synthesis with random hexamer primers and SuperScript III reverse transcriptase (Invitrogen). SYBR Premis Ex Taq (TaKaRa) and gene-specific primers provided for the Primer Express Software v3.0.1 (Life technologies) were used for the reactions. Quantitative RT-PCR (qRT-PCR) analyses were performed using 7500 Fast Real-Time PCR systems (Applied Biosystems). The relative expression levels in each transcript were obtained by normalization to the actin gene (AB181991). The gene-specific primer sets were listed in S1 Table.

## Results

### Continuous monitoring of shoot biomass production reveals differences in growth responses to salinity between the bread wheat cultivars

**Fig 1:**
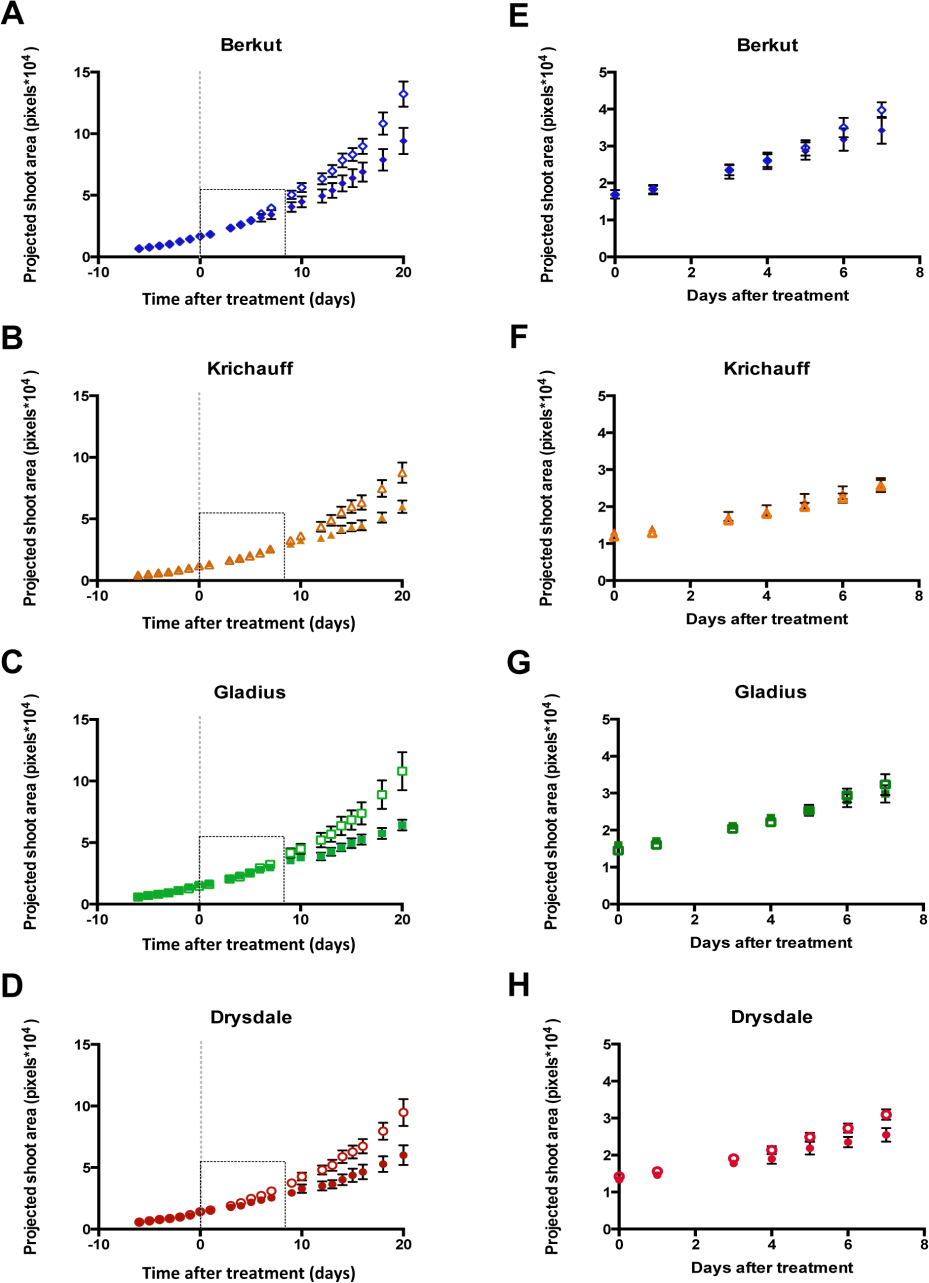
Sequential monitoring of shoot biomass under conditions of salinity stress. Quantification of growth through time under conditions of salinity stress of (A) Berkut (blue diamonds, SE, n = 6), (B) Krichauff (orange triangles, SE, n = 6,), (C) Gladius (green squares, SE, n = 4) or (D) Drysdale (red circles, SE, n = 4). Cultivars were treated with RO water (open symbols) or 100 mM NaCl (filled symbols) at the emergence of the fourth leaf (vertical line indicates day 0 or day of treatment). Projected shoot area (pixels) was determined by image analysis from digital images taken with RGB cameras. Exponential curves were fitted to data from day 0 to day 7 after treatment (rectangle within graphs A to D) to calculate relative growth rates for (E) Berkut, (F) Krichauff, (G) Gladius and (H) Drysdale seedlings under control and saline conditions.

Four commercial bread wheat cultivars were investigated for variation in salinity tolerance: Berkut, Krichauff, Gladius and Drysdale. Previous studies indicate that the doubled-haploid (DH) mapping population from a cross between Berkut and Krichauff were analyzed as salinity tolerance (ST) quantitative trait loci (QTL) to improve the grain yield-independent ST in field [24, 25]. A high-throughput phenotyping system was used to measure the growth of these bread wheat cultivars, and to quantify the effects of salinity on their growth. To evaluate the shoot ion independent component of salinity stress, the relative growth rate (RGR) of plants was estimated using the images of the projected shoot area for 8 days after treatment, and exponential growth curves fitted through the projected shoot area data [21, 26, 27]. For the relatively short time period of this experiment, the assumption of exponential growth was found to be a satisfactory approximation (Fig 1 and S3 Fig).

**Fig 2:**
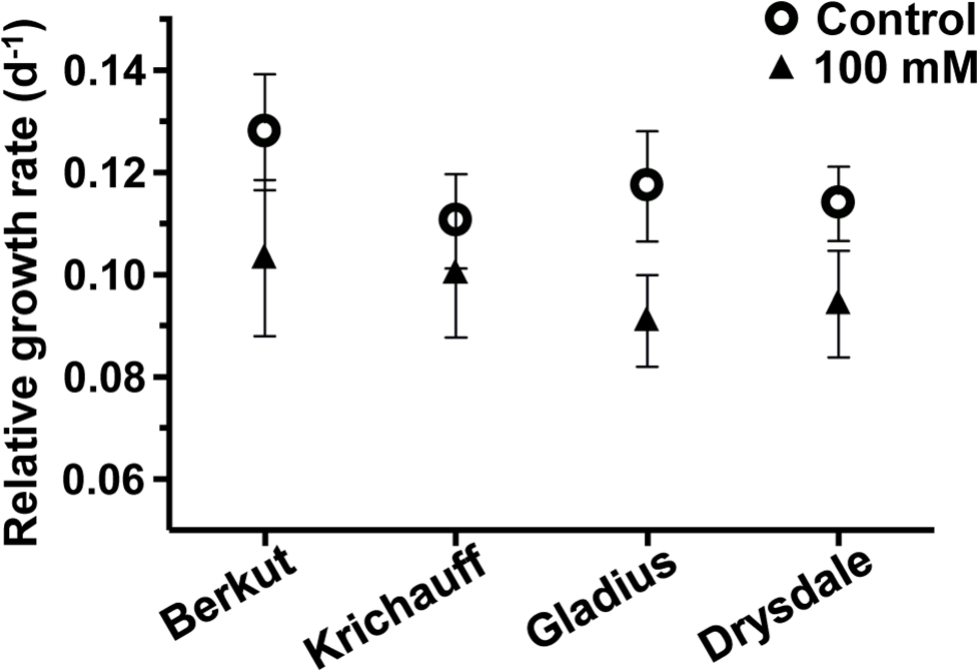
Relative growth rates (RGRs) of seedlings of Berkut, Krichauff, Gladius and Drysdale over 0 to 7 days grown with no added NaCl (circles) or treated with 100 mM NaCl (triangles). (SE, n = 6 for Berkut and Krichauff, n = 4 for Gladius and Drysdale). RGRs of treated plants were significantly difference to RGRs of control plants (2 way ANOVA, p = 0.025).

Over the time course of the experiments, Berkut showed the highest mean RGR under control conditions (Figs 1 and 2). Treatment with 75 mM and 100 mM NaCl caused little reduction in RGR for both Drysdale and Krichauff, and a greater reduction in RGR under salinity was observed for Gladius and Berkut (Fig 2 and S3E Fig). These differences can be best summarized by calculating the salinity tolerance index (STI: Table 1), which indicated that Krichauff had the highest salinity tolerance under those salinity stress conditions (strictly, the greatest ability to maintain RGR over this time period in saline conditions relative to non-saline conditions).

**Table 1:** Salt tolerance index (STI) for the four bread wheat genotypes, calculated from the RGRs presented in Fig 2 and S3E Fig.

Genotype	STI	
	75 mM /Cont.	100 mM /Cont.
Berkut	0.877	0.808
Krichauff	0.953	0.908
Gladius	0.846	0.776
Drysdale	0.900	0.828

Genotype x Salt, *p* < 0.05.

### Cluster analysis of variation in gene expression in leaf sheaths with respect to shoot growth and salinity stress tolerance

Microarray profiling of gene transcripts from leaf sheaths was performed to identify genes responding to the early stages of salinity stress. We selected the treatment of wheat plants with 100 mM NaCl for microarray analysis because the treatment with 100 mM NaCl affected the further widening of the difference of STI in comparison with the treatment of wheat with 75 mM NaCl among four cultivars (Table 1). For each cultivar, analysis was performed for both control and 100 mM NaCl treated plants on days 1, 2, and 3 after treatment. Integrated gene expression profiles for the treatment periods indicated hierarchical clustering side-by-side with control and moderate salinity stress conditions in each cultivar (S4A Fig). A 3D principal components analysis (PCA) revealed that the gene expression clusters for these cultivars differ significantly from each other even under control conditions (S4B Fig). Moreover, the large differences in expression of genes were observed between cultivars in samples from salt stressed plants, indicating that the gene expression profiles are different among different cultivars under both control and salinity stress conditions.

**Fig 3:**
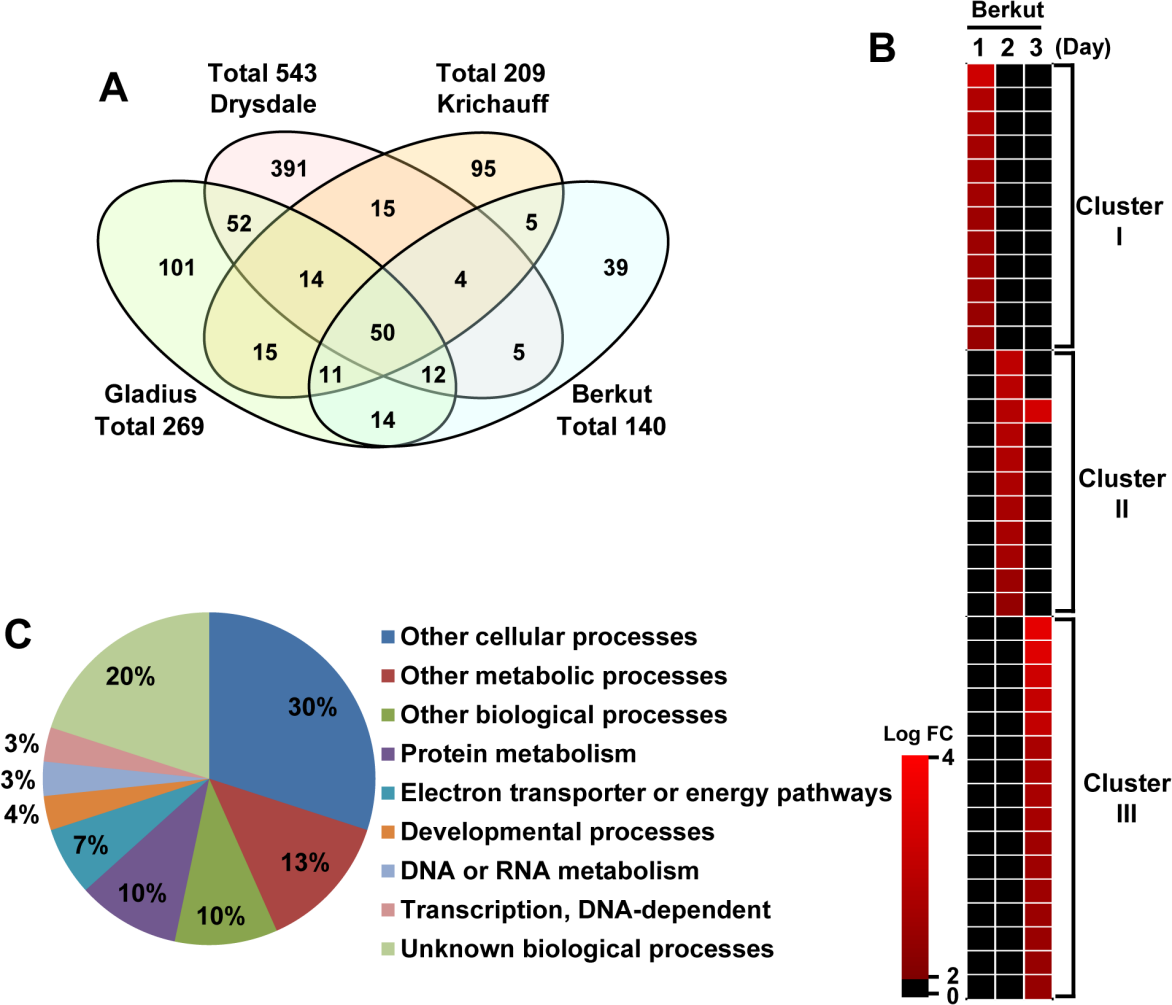
Gene expression pattern for the four cultivars under control conditions. (A) Venn diagrams showing a five-fold or greater difference in expression with all possible regressions during the first 3 days of growth under control conditions compared with the starting point (day 0) of the experiments. (B) Heat maps indicating intensity of gene expression in Berkut. Log FC = log_2_ (the signal intensity under saline conditions / the signal intensity under control conditions). (C) Analysis of the gene ontology of 39 genes upregulated only in Berkut under control conditions. Functional categorizations by annotation were shown as gene ontology of biological process.

Although the patterns of leaf sheath transcriptome regulation varied sharply among the cultivars, an attempt was made to identify common responsive genes governing shoot biomass development and salinity tolerance. We focused first on RGRs under control conditions. Berkut showed the highest mean RGR under control conditions in both experiments (Fig 2 and S3 Fig). Comparative gene expression analysis revealed that 39 genes were upregulated only in Berkut during the first 3 days of growth under control conditions compared with the starting point (day 0) of the experiments (Fig 3A).

The timing of expression of the 39 genes exhibited three tendencies. The first, shown as cluster I, comprises the early response that occurred within 24 h after control conditions (Fig 3B). Cluster II comprises expressed genes in the second day under control conditions. Cluster III includes the late response that occurred on the 3^rd^ day. All 39 genes were searched against the annotated genome datasets of both *Arabidopsis* and rice (TAIR 10 and RAP-DB) to identify their function. A comparative gene annotation analysis of wheat with rice and *Arabidopsis* indicated that most homologous genes have known functions (Table 2). Functional category classification using the gene ontology (GO) term revealed that most of the 39 genes were assigned to cellular and metabolic processes, developmental processes, and cell organization and biogenesis, suggesting that Berkut has a specific ability to maintain a high growth rate in the early stages of growth by transcription of particular genes involved in plant growth (Fig 3C).

**Table 2:** Differentially expressed genes during biomass accumulation under control conditions in Berkut.

Cluster				Log fold-change				
	Probe Name ^a^	Locus ^b^		Berkut		Load ID ^c^	AGI code ^d^	Descriptions ^e^
			1 Day	2 Day	3 Day			
Cluster I	A_99_P412102	Y17386	3.24			Os03g0283200		Similar to IN2-1 protein
	A_99_P432617	CJ685030	2.76			Os07g0631100		Protein of unknown function DUF701, zinc-binding putative family protein
	A_99_P240161	CA656262	2.69			Os10g0191300		Similar to PR-1a pathogenesis related protein (Hv-1a) precursor
	A_99_P240196	CA666553	2.58			Os10g0191300		Similar to PR-1a pathogenesis related protein (Hv-1a) precursor
	A_99_P313761	CJ585429	2.55			Os01g0893400		BTB domain containing protein
	A_99_P330831	JP215434	2.55			Os09g0497100	At4g25880	Pumilio 6
	A_99_P039764	CA682812	2.48					Unknown
	A_99_P422587	TC382749	2.45					Unknown
	A_99_P240171	CA605668	2.44			Os10g0191300		Similar to PR-1a pathogenesis related protein (Hv-1a) precursor
	A_99_P082355	DR737985	2.41			Os01g0644200		Conserved hypothetical protein
	A_99_P420152	CK213961	2.38			Os01g0971700		Streptomyces cyclase/dehydrase family protein
	A_99_P570547	CJ593115	2.37			Os11g0498600		Similar to HVA22 protein
Cluster II	A_99_P150212	CJ832771		2.97				Unknown
	A_99_P535012	CD929060		2.90		Os06g0535400		Similar to E3 ubiquitin ligase EL5
	A_99_P445077	CJ631312		2.85	3.32	Os04g0411200		Conserved hypothetical protein
	A_99_P307246	CJ561077		2.81		Os10g0489500		Terpenoid cylases
	A_99_P341696	AK331429		2.75		Os12g0559200		Lipoxygenase
	A_99_P261956	TC373079		2.70				Unknown
	A_99_P050529	CA627809		2.63		Os04g0119000		Conserved hypothetical protein
	A_99_P542092	CA626603		2.63				Unknown
	A_99_P444042	BJ233618		2.58		Os12g0274700	At5g38420	Ribulose bisphosphate carboxylase
	A_99_P076050	CK172017		2.45		Os02g0784700	At1g53750	Regulatory particle triple-A 1A
	A_99_P374642	HP627383		2.33		Os09g0533300		Phosphoesterase At2g46880 family protein
Cluster III	A_99_P518712	BJ282418			3.61	Os02g0662000		RCc3 protein
	A_99_P000496	AF174433			3.51	Os05g0519700	At1g74310	Heat shock protein 101
	A_99_P195793	DQ512350			3.22	Os08g0112700		Similar to TAGL12 transcription factor
	A_99_P479287	AJ604304			3.09	Os03g0266900		Low molecular mass heat shock protein Oshsp17.3
	A_99_P052261	CA641120			3.05			Unknown
	A_99_P074855	CK155862			2.66			Unknown
	A_99_P523067	CV779736			2.65	Os05g0407100		Four F5 protein family protein
	A_99_P005701	CK207563			2.64	Os03g0582000		Formiminotransferase
	A_99_P325241	AK335952			2.61	Os03g0756200	At1g69270	Receptor-like protein kinase 1
	A_99_P392277	BQ806868			2.51	Os03g0756200		Protein kinase-like domain containing protein
	A_99_P175099	DR740945			2.48	Os03g0103200		Similar to Physical impedance induced protein
	A_99_P479697	CD454493			2.48	Os02g0716500		Similar to Delta-12 fatty acid desaturase
	A_99_P536458	DR732512			2.43	Os11g0455500	At4g13940	HOG1, SAHH1
	A_99_P557092	BQ901904			2.41			Unknown
	A_99_P294126	JP220179			2.38	Os04g0584800	At5g65960	GTP binding
	A_99_P032024	CJ887501			2.36			Unknown

Listed are genes with expression increased more than five-fold in Berkut compared with other cultivars under control conditions. *p* values of <0.01 were included.

^a^ Probe name as given by Agilent 44K wheat gene expression array (Design ID: 022297).

^b^ Locus indicates GenBank accession.

^c^ Load ID as given by RAP-DB.

^d^ AGI, Arabidopsis Genome Initiative.

^e^ Description as given by The Institute for Genomic Research database.

**Fig 4:**
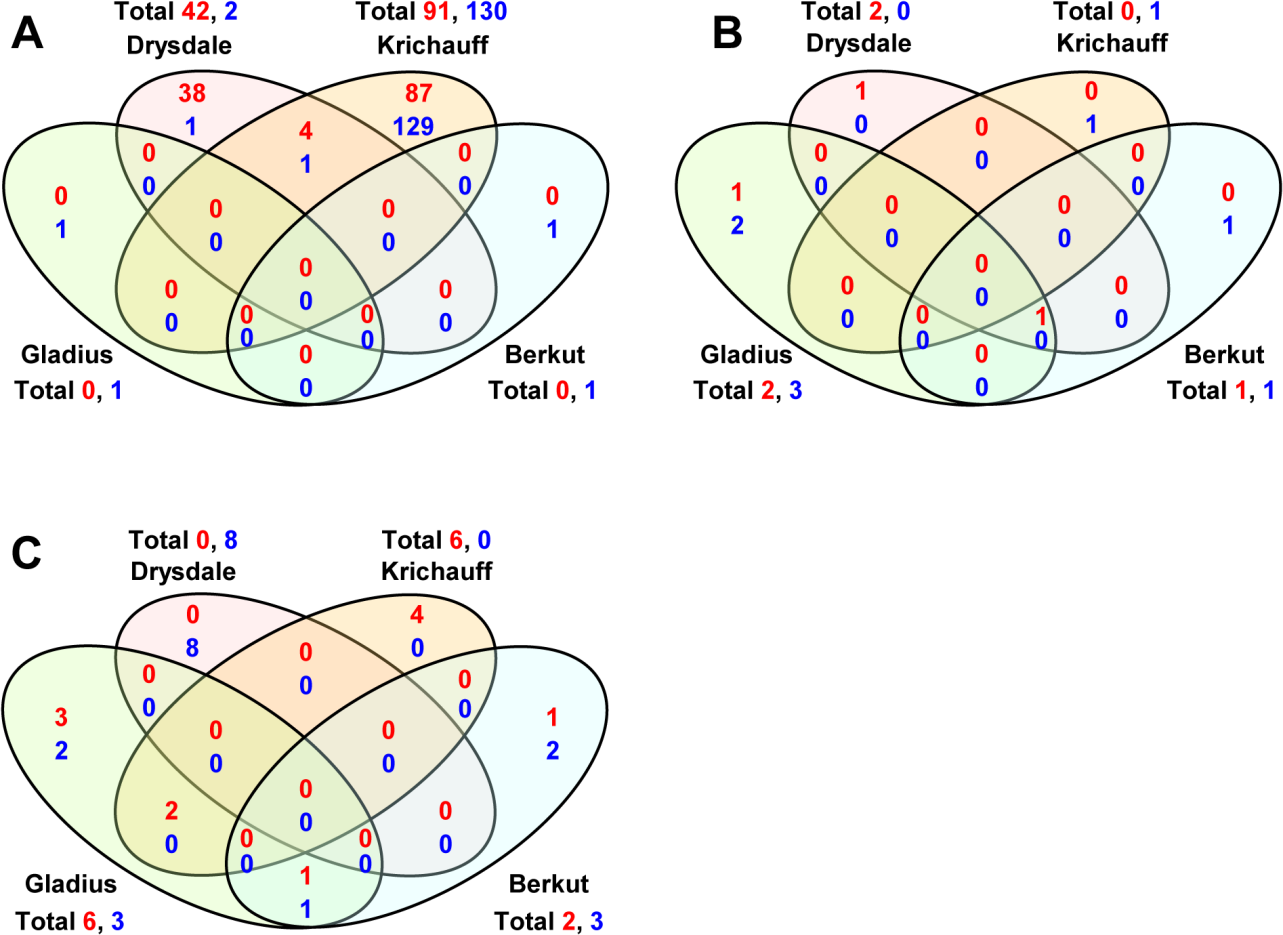
Venn diagram analyses of genes regulated in all four cultivars under salinity conditions (100 mM NaCl). (A–C) Venn diagrams showing a five-fold or greater difference in expression at 1 (A), 2 (B) or 3 (C) days after NaCl treatment. Red numeric characters show the number of upregulated genes. Blue numeric characters show the number of downregulated genes.

**Fig 5:**
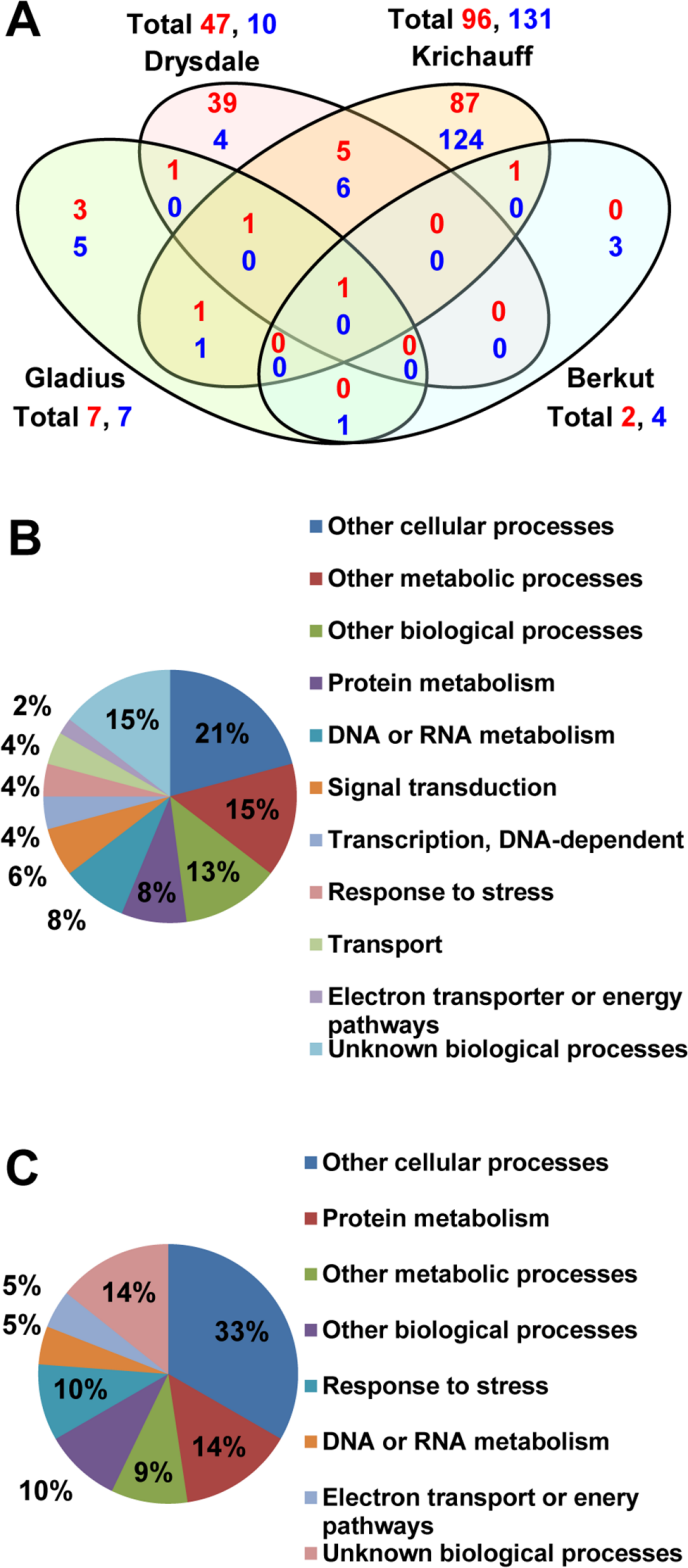
Gene expression pattern for four cultivars under conditions of salinity stress. (A) Venn diagrams showing a five-fold or greater difference in expression with all possible regressions under saline conditions (100 mM NaCl). Red numeric characters show the number of upregulated genes. Blue numeric characters show the number of downregulated genes. (B) Analysis of the gene ontology of 48 genes increased only in Krichauff under saline conditions. Functional categorizations by annotation were shown as gene ontology of biological process. (C) Analysis of the gene ontology of 21 genes increased only in Drysdale under saline conditions.

Drysdale and Krichauff showed higher STI with the 100 mM NaCl treatment in comparison with Gladius and Berkut (Table 1), and they showed specific temporal variations in gene expression in response to salinity stress (Fig 4). Most upregulated and downregulated genes were expressed within 24 h of salinity treatment in both cultivars (Fig 4A). Of the 47 genes in Drysdale and 96 genes in Krichauff that were upregulated under salinity stress compared with control (RO water) conditions, 42 (89.4%) and 91 (94.8%), respectively, were observed to be upregulated one day after treatment. However, only five genes were similarly regulated in both cultivars (Fig 5A and Table 3). In contrast, the more salt sensitive Gladius and Berkut varieties did not exhibit dramatic changes in the numbers of upregulated genes during the three days after stress treatment, with only about 10 genes upregulated by mild salinity stress in each of the varieties. The same tendency was observed in the gene sets which were downregulated under mild salinity stress conditions (Figs 4 and 5A and S2 Table).

**Table 3:** Differentially expressed genes during salinity stress in Drysdale and Krichauff when compared to Berkut and Gladius.

Probe Name ^a^		Log fold-change		Log fold-change				
	Locus ^b^	Drysdale		Krichauff		Load ID ^c^	AGI code ^d^	Descriptions ^e^
		1 Day	2 Day	1 Day	3 Day			
A_99_P457852	CA728141	4.04		10.91		Os04g0691600 *	At1g79850	30S ribosomal protein S17
A_99_P144338	CJ851704	3.32		2.51		Os02g0190500	At1g16150 *	WALL ASSOCIATED KINASE-LIKE 4
A_99_P069515	CD491253	2.74			2.49			Unknown
A_99_P146292	CK153204	2.54		8.38				Unknown
A_99_P481317	CD877401	2.48		6.31		Os03g0307200		Nicotianamine synthase 2

Listed are genes where expression was increased more than five-fold in Drysdale and Krichauff compared with other cultivars under saline conditions. *p* values of <0.01 were included. Asterisk indicates stress- and/or abscisic acid treatment-inducible gene in rice and *Arabidopsis*.

^a^ Probe name as given by Agilent 44K wheat gene expression array (Design ID: 022297).

^b^ Locus indicates GenBank accession.

^c^ Load ID as given by RAP-DB.

^d^ AGI, Arabidopsis Genome Initiative.

^e^ Description as given by The Institute for Genomic Research database.

Krichauff consistently showed the highest STI compared with the other three varieties under moderate salinity stress conditions (Table 1). Transcriptome analysis showed that 87 genes were upregulated only in the leaf sheaths of Krichauff under salinity conditions (Fig 5A). The annotated nucleotide database of RAP-DB contained rice homologs of 48 of the 87 genes (Table 4). Using gene ontology analysis, most of the 48 genes were assigned as encoding proteins involved in cellular and metabolic processes (Fig 5B). The Krichauff-specific genes were accordingly classified as genes associated with the persistence of vigorous growth under mild salinity stress conditions.

**Table 4:** Differentially expressed genes during biomass accumulation under saline conditions in Krichauff.

Probe Name ^a^		Log fold-change				
	Locus ^b^	Krichauff		Load ID ^c^	AGI code ^d^	Descriptions ^e^
		1 Day	3 Day			
A_99_P200001	DR740066	8.14		Os05g0477900 *		Similar to Nonspecific lipid-transfer protein 1 (LTP 1)
A_99_P264756	CA639657	7.72		Os02g0761900		Dimethylmenaquinone methyltransferase family protein
A_99_P554047	CA728814	7.66		Os03g0248300 *		Similar to Cytochrome P450
A_99_P324811	AK335197	7.41		Os08g0158500		Similar to Stomatin-like protein
A_99_P448682	CK205818	7.38		Os08g0157500		Similar to Caffeic acid 3-O-methyltransferase
A_99_P012199	AK334460	7.07				Unknown
A_99_P101440	BJ305619	6.77		Os01g0970500		Similar to Transcription factor IIA small subunit
A_99_P495222	CJ560501	6.66		Os02g0616100		Conserved hypothetical protein
A_99_P474007	TC418856	6.63				Unknown
A_99_P177389	U32429	6.55				Unknown
A_99_P166449	HP637526	6.52		Os09g0420700		Similar to Cox17p
A_99_P062343	CV774252	6.44				Unknown
A_99_P097185	CD876160	6.33				Unknown
A_99_P229581	CK159562	6.11				Unknown
A_99_P100730	BT009029	6.04		Os06g0285200 *	At1g21970 *	LEC1, EMB 212, EMB212, NF-YB9
A_99_P299921	CV780407	5.99				Unknown
A_99_P546222	CJ855445	5.97				Unknown
A_99_P023214	CO349141	5.52		Os02g0580600 *		Similar to Dimethylaniline monooxygenase-like protein
A_99_P007696	AK333494	5.41				Unknown
A_99_P000146	JP207204	5.27		Os03g0610800		Similar to Protein zx
A_99_P340071	JP221353	5.12				Unknown
A_99_P154087	CJ931367	5.03		Os09g0570200		Zinc finger, C2H2-type domain containing protein
A_99_P004166	HP629655	5.01				Unknown
A_99_P123925	CV764156	4.99			At5g57080	Unknown protein
A_99_P184762	CJ726963	4.97		Os10g0571200		Similar to Pyruvate kinase isozyme G
A_99_P100350	DR741521	4.96				Unknown
A_99_P352331	AK335348	4.88		Os11g0689100		Disease resistance protein family protein
A_99_P464282	DR741256	4.85		Os01g0963600		ABA/WDS induced protein family protein
A_99_P059005	CJ902984	4.66		Os06g0727900	At4g37420	Domain of unknown function (DUF23)
A_99_P147347	DR732549	4.60		Os10g0174800	At1g16150 *	Wall associated kinase-like 4
A_99_P150187	CJ832676	4.59				Unknown
A_99_P147622	DR740974	4.52				Unknown
A_99_P000296	U32430	4.51		Os01g0330200		Peptidase C1A, papain family protein
A_99_P466377	CV761504	4.47		Os11g0454300 *		Similar to Water-stress inducible protein RAB21
A_99_P119649	BE604619	4.43		Os07g0585500		Conserved hypothetical protein
A_99_P457002	CJ703861	4.43				Unknown
A_99_P498792	CD881238	4.35		Os08g0430100		Conserved hypothetical protein
A_99_P245941	CJ585664	4.26		Os01g0941200		Similar to Glucan endo-1,3-beta-glucosidase GII precursor
A_99_P198406	AK332908	4.18		Os08g0157500	At5g54160	O-methyltransferase 1
A_99_P467132	CJ620076	4.06		Os08g0565800		Similar to Glutaredoxin
A_99_P173954	DQ872405	4.04				Unknown
A_99_P031359	BF482287	3.98				Unknown
A_99_P121369	CJ628798	3.90		Os09g0565400		Lipoprotein, type 6 family protein
A_99_P397357	CA648273	3.89				Unknown
A_99_P145321	CJ954125	3.77				Unknown
A_99_P120899	CJ718634	3.67				Unknown
A_99_P160052	AK330678	3.61		Os01g0360600		Dephospho-CoA kinase family protein
A_99_P013754	BJ290482	3.54				Unknown
A_99_P072545	CD862488	3.48				Unknown
A_99_P119539	CJ703294	3.38				Unknown
A_99_P487867	CK163549	3.37		Os01g0975900 *	At3g26520	Tonoplast intrinsic protein 2
A_99_P440802	CK166064	3.35		Os01g0149800	At3g09390 *	Metallothionein 2A
A_99_P006996	DN829327	3.32				Unknown
A_99_P466182	DR740299	3.30				Unknown
A_99_P001301	CA730969	3.22		Os04g0581800		Heavy metal transport
A_99_P072965	CD886273	3.21		Os01g0713700		Similar to Rev interacting protein mis3-like
A_99_P020974	CJ638392	3.17				Unknown
A_99_P517167	CO349122	3.16				Unknown
A_99_P020754	AY234333	3.11		Os04g0664500		Similar to Agmatine coumaroyltransferase
A_99_P132885	CJ696327	3.11				Unknown
A_99_P129600	CJ654400	3.08				Unknown
A_99_P463762	CJ631087	2.96				Unknown
A_99_P443267	CD452924	2.95		Os01g0128200 *		Similar to Nuclease I
A_99_P166107	JF288936	2.94		Os01g0975300		Similar to Typical P-type R2R3 Myb protein
A_99_P389992	AL816502	2.94				Unknown
A_99_P228041	CV778105	2.94		Os11g0592000 *		Similar to Barwin
A_99_P071940	BT009002	2.90		Os03g0850700		Phosphatidylinositol phosphatidylcholine transfer protein sec14
A_99_P571917	CD931265	2.89		Os01g0121700		ABC transporter related domain containing protein
A_99_P482852	BJ250291	2.84		Os06g0115500	At3g49010	Breast basic conserved 1
A_99_P153187	CJ883271	2.78		Os10g0467000 *		Conserved hypothetical protein
A_99_P557422	AK331284	2.76		Os08g0385900		Similar to Transformer-2-like protei
A_99_P534162	AL830283	2.75		Os06g0139700		Conserved hypothetical protein
A_99_P119479	CJ671693	2.68		Os02g0803700		Similar to 26S protease regulatory subunit 6A homolog
A_99_P349031	AK336292	2.65				Unknown
A_99_P152982	CJ876786	2.65				Unknown
A_99_P421547	AK332152	2.63		Os10g0397400	At3g19820	Cell elongation protein / DWARF1
A_99_P348706	HP626963	2.63		Os10g0464000	At5g62740	PHB domain-containing membrane-associated protein family
A_99_P091705	HP624059	2.62		Os05g0107600	At4g39850 *	Peroxisomal ABC transporter 1
A_99_P125805	CJ697178	2.58		Os08g0461100		Leucine-rich repeat 2 containing protein
A_99_P000206	CK214300	2.53				Unknown
A_99_P089580	CJ777894	2.41				Unknown
A_99_P113810	CJ654682	2.41				Unknown
A_99_P495952	CJ595937	2.40		Os04g0347100		Conserved hypothetical protein
A_99_P149362	CJ850274	2.33				Unknown
A_99_P229231	JP207400		2.86	Os03g0663800		Cupin region domain containing protein
A_99_P431867	CK163906		2.84	Os03g0663800		Cupin region domain containing protein
A_99_P397607	CD900068		2.76			Unknown

Listed are genes where expression was increased more than five-fold in Krichauff compared with other wheat cultivars under salinity stress conditions. *p* values of <0.01 were included. Asterisk indicates stress- and/or abscisic acid treatment-inducible genes in rice and *Arabidopsis*.

^a^Probe name as given by Agilent 44K wheat gene expression array (Design ID: 022297).

^b^Locus indicates GenBank accession.

^c^Load ID as given by RAP-DB.

^d^AGI, Arabidopsis Genome Initiative.

^e^Description as given by The Institute for Genomic Research database.

Drysdale showed second highest STI compared with the other three varieties (Table 1). GO analysis showed that rice homologs of 21 of the 39 genes which were upregulated only in Drysdale were also classified as genes associated with the cellular, metabolic and biological processes (Fig 5C and Table 5).

**Table 5:** Differentially expressed genes during biomass accumulation under saline conditions in Drysdale.

Probe Name ^a^		Log fold-change				
	Locus ^b^	Drysdale		Load ID ^c^	AGI code ^d^	Descriptions ^e^
		1 Day	2 Day			
A_99_P371582	HP634553	3.68				Unknown
A_99_P116810	CJ666835	3.45				Unknown
A_99_P190337	CK198823	3.42		Os09g0240500		Similar to Sulfate transporter 4.1
A_99_P407832	CK210016	3.34		Os12g0624000	At3g03780	Methionine synthase 2
A_99_P033359	CK211257	3.32		Os01g0823600		Conserved hypothetical protein
A_99_P158277	CK213509	3.15		Os11g0453900 *		Dehydrin RAB 16D
A_99_P142173	CJ896691	3.13				Unknown
A_99_P341226	AK334232	3.06				Unknown
A_99_P480802	CK207636	3.06		Os02g0135500		Protein of unknown function DUF563 family protein
A_99_P019814	CD454940	3.04		Os06g0325900 *		Similar to Bx2-like protein
A_99_P062623	DR737352	2.95		Os06g0729400 *		Similar to gibberellin-regulated protein 2 precursor
A_99_P124640	CK200790	2.94				Unknown
A_99_P355916	CB307808	2.92				Unknown
A_99_P176586	CA484022	2.81		Os05g0592600		Initiation factor 2 family protein
A_99_P110605	DR734963	2.75				Unknown
A_99_P293566	CD869841	2.73				Unknown
A_99_P080740	CN009859	2.69				Unknown
A_99_P162972	DR740359	2.69		Os04g0448800	At3g48850	PHT3;2 (Phosphate transporter)
A_99_P364411	EB513470	2.68		Os02g0650300	At5g53550 *	YSL3
A_99_P475337	CK203004	2.68				Unknown
A_99_P020624	CV771491	2.66		Os11g0524300		Protein of unknown function DUF1001 family protein
A_99_P611632	EF561285	2.52				Unknown
A_99_P365356	AK332187	2.48				Unknown
A_99_P139130	CK161730	2.48				Unknown
A_99_P012784	BJ283765	2.46				Unknown
A_99_P015049	CJ827289	2.45				Unknown
A_99_P486197	CD870476	2.44		Os03g0737000 *		CBS domain-containing protein
A_99_P002001	BE402442	2.44				Unknown
A_99_P514542	CJ630561	2.40		Os05g0539500		Esterase/lipase/thioesterase domain containing protein
A_99_P472107	CV782599	2.39		Os07g0112800	At1g69410 *	ELF5A-3 (Eukaryotic elongation factor 5A-3)
A_99_P069010	CD490965	2.38		Os04g0661200		Protein of unknown function DUF941 family protein
A_99_P467927	CA630209	2.37		Os03g0704000		Similar to 30S ribosomal protein S13
A_99_P601087	AY831779	2.36				Unknown
A_99_P293761	CK193081	2.36		Os06g0730800		Cwf18
A_99_P022159	BQ162167	2.35		Os10g0567500		Intron maturase, type II family protein
A_99_P134895	CV776886	2.35		Os02g0227000		Protein of unknown function UPF0054 family protein
A_99_P058076	CJ778180	2.33				Unknown
A_99_P147332	DR732448	2.32		Os04g0244800 *		Heavy metal transport
A_99_P259546	CK215676		2.69	Os11g0498600		Similar to HVA22 protein

Listed are genes with expression increased more than five-fold in Drysdale compared with other wheat cultivars under saline conditions. *p* values of <0.01 were included. Asterisk indicates stress- and/or abscisic acid treatment-inducible genes in rice and *Arabidopsis*.

^a^Probe name as given by Agilent 44K wheat gene expression array (Design ID: 022297).

^b^Locus indicates GenBank accession.

^c^Load ID as given by RAP-DB.

^d^AGI, Arabidopsis Genome Initiative.

^e^Description as given by The Institute for Genomic Research database.

**Fig 6:**
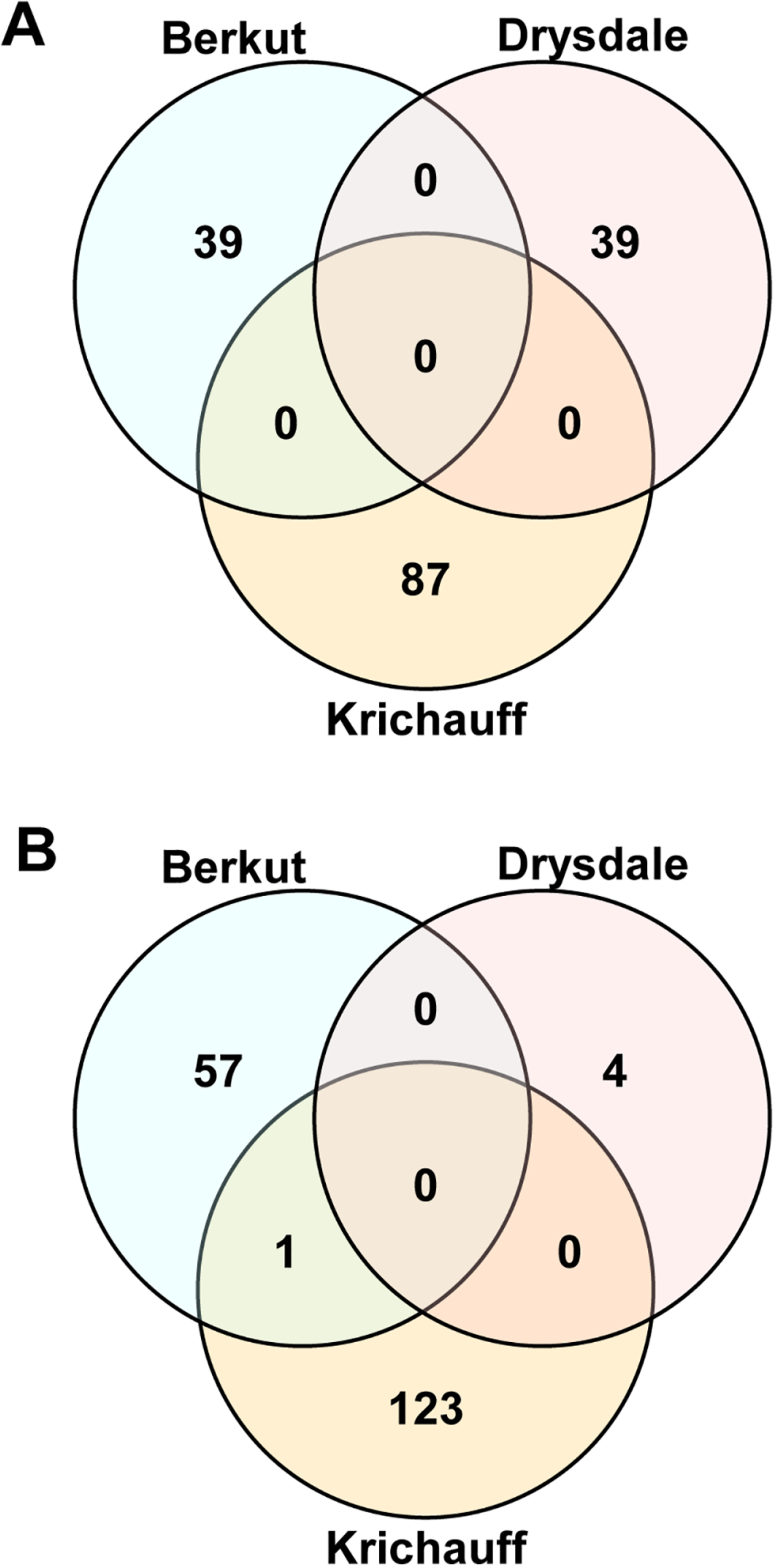
Comparative analysis of gene expression among regulated genes in Berkut under control conditions, in Krichauff under saline conditions and in Drysdale under saline conditions (100 mM NaCl). (A) Venn diagrams show the comparative analysis of upregulated gene expression associated with each specific physiological trait. (B) Venn diagrams show the comparative analysis of downregulated gene expression associated with each specific physiological trait.

To identify differences between gene expression profiles associated with shoot biomass development under control and saline conditions, gene sets showing expression only in Berkut under control conditions (Table 2 and S3 Table), only in Krichauff under saline conditions (Table 4 and S4 Table) and only in Drysdale under saline conditions (Table 5 and S5 Table) were compared. However, the Venn diagram shows no overlap among the 39 genes upregulated in Berkut, the 87 genes upregulated in Krichauff and the 39 genes upregulated in Drysdale (Fig 6A). The 58 genes downregulated in Berkut, the 124 genes downregulated in Krichauff and the 4 genes downregulated in Drysdale also showed no overlap (Fig 6B).

Quantitative RT-PCR (qRT-PCR) was performed to validate the gene expression profiles obtained by microarray analysis. We randomly selected several genes which were expressed in either control or 100 mM NaCl treated plants (Figs 3A and 5A). The scatter plots showed that the results of the qRT-PCR analysis correlate highly with those of the microarray analysis (S5 Fig). The determination coefficient (*R* ^2^ = 0.8418) indicates that the microarray analysis provides the reliable data to identify genes responding to the early stages of salinity stress.

### Comparative analysis of gene expression profiles of bread wheat with those of Arabidopsis and rice revealed physiological differences in salinity stress responses

To determine whether the upregulated genes identified only in Krichauff and only in Drysdale under salinity treatment are also expressed in other plants, the expression profiles of the homologous genes in *Arabidopsis* and rice were examined using public gene expression databases (Tables 3–5). Using the *Arabidopsis* eFP Browser and Rice Expression Profile Database (RiceXPro), it was possible to determine the expression patterns of the 48 homologous of the upregulated Krichauff genes in *Arabidopsis* or rice after they had been exposed to either abiotic stress or hormone treatment. Only 12 rice and/or *Arabidopsis* homologs showed a tendency to respond to salinity stress and/or abscisic acid-inducible expression. In the 21 homologous genes upregulated only in Drysdale, the 7 rice and/or *Arabidopsis* homologs showed a tendency to response to stress-inducible expression. These results suggest that the gene expression responses in commercial bread wheat under mild salinity stress may be different from those in *Arabidopsis* and rice under severe salinity stress, and that bread wheat varieties with high RGR and high salinity tolerance have a unique transcriptome pattern.

## Discussion

Several genes resolved in our microarray results have been reported as regulators of stress tolerance in other plants. *Dehydrin*/*responsive to ABA* (*RAB*), the homolog of CK213509 and CV761504, is a well-known family of hydrophilic proteins, and strongly responsive to water stresses and ABA treatment [28]. The *cell wall-associated receptor kinase* (*WAK*) and *WAK-like kinase* (*WAKL*) gene family are involved in biotic and abiotic stress responses and are required for cell elongation and development [29, 30]. The expression of *WAKL4*, the homolog of CJ851704, is induced by several mineral responses including to salt. This finding indicates that these membrane-to-cytoplasm communication signals are present in wheat under salinity stress as well as in other plants [31]. Lipid transfer protein (LTP) is involved in stress responses, and overexpression of LTP mediates salinity tolerance [32]. The *LTP* homolog AK332187 is expressed under salinity stress in stress-resistant wheat cultivars, suggesting a process of regulation similar to that for equilibrating metabolite accumulation. In addition, the results from the temporal microarray analysis performed in our study indicate that the experimental system properly captured the early response of gene expressions during the osmotic phase.

Our analysis sheds light on the unique molecular mechanisms of gene expression in commercial bread wheat under mild salinity conditions. The results from the 3D PCA revealed a wide diversity in leaf sheath transcriptome profiles for the four commercial cultivars studied (S4 Fig). Our results indicate that the salt stress-inducible gene sets generated during the osmotic phase are distinct between the four cultivars (Fig 5). The comparative analysis of variation in gene expression in relation to physiological function revealed the causal components associated with development of biomass and tolerance to mild salinity stress (Fig 6). In addition, most genes expressed in the leaf sheath of the four bread wheat cultivars tested do not correspond with salinity stress-inducible genes described in other well-characterized plants, such as rice and *Arabidopsis* (Tables 3–5). These results suggest that genetic variation resulting from selection during breeding exerts a strong influence on the development of genome architecture and adaptation to salinity conditions.

An integrated analysis of the transcriptomes and epigenomes in two rice subspecies revealed a high correlation of allelic bias of epigenetic modification with gene expression [33]. Bioinformatics approaches have shown that *cis*-regulatory mutations are the major source of evolutionary innovations and that these alterations lead to changes in gene regulatory information and phenotypic defects [34]. A recent genome-wide association study identified sequence variants between cultivars and wild rice in promoter regions [35]. Comparative transcriptome analysis between domesticated and wild tomatoes revealed that thousands of gene expression patterns were different and that most of them were associated with environmental responses and stress tolerance [36]. These studies are consistent with the present study in suggesting that genomic variation affects salinity tolerance through changes in gene expression.

In particular, gene ontology analysis revealed that the genes involved in a number of cellular and metabolic processes were more upregulated for shoot biomass development than stress response genes (Fig 5), indicating that salinity-tolerant cultivars have the ability of improving biomass development with the alterations in metabolism-mediated gene expressions compared with stress tolerance under mild salinity conditions. For Australian bread wheat, improvements in salinity tolerance and the ability to continue to increase biomass under mild salinity stress conditions are particularly important. The breeding of salinity tolerant cultivars can exploit this study for knowledge of an understanding of sequence variants associated with the domestication of salinity tolerant Australian bread wheat.

In this study, we evaluated, under control and mild saline conditions in a greenhouse, the physiological differences between four commercial bread wheat cultivars. The high-throughput phenotyping system at The Plant Accelerator of the Australian Plant Phenomics Facility allowed the measurement through time of shoot biomass, thus enabling calculations of RGR (Fig 1, S3 Fig and Table 1). This phenotyping analysis in The Plant Accelerator was more quantitative than is practically achievable in most other experiments in both controlled conditions and in the field. Therefore, the phenotyping system facilitates the analysis of useful germplasm using detailed molecular analyses, including microarrays. This study established that a high-throughput greenhouse phenotyping system, such as The Plant Accelerator, allows a powerful approach, combining physiological trait evaluation with molecular analysis of gene expression in bread wheat cultivars under mild saline conditions.

Our microarray analysis indicated that changes in the expression of salt stress-inducible genes reproducibly decreases within 2 days (Fig 4), suggesting that rapidly-induced gene expression, occurring shortly after the perception of stress, has a significant influence on adaptation to mild salinity conditions in the early osmotic phase. When combined with previous studies on Na^+^ and Cl^-^ ions accumulation which gradually occurs under stress conditions, these results indicate that two phases, osmotic and ionic, are involved in responses and adaptation to mild salinity stress conditions in the bread wheat cultivars tested, and that the physiological characteristics of each phase are different from the perspective of acquiring salinity tolerance. Combined analysis of relative shoot growth, stress tolerance and Na^+^ exclusion constitutes a detailed analysis of mechanisms underlying salinity tolerance in both the osmotic and ionic phases during mild salinity treatment.

In conclusion, our integrated systematic analyses of phenomics and transcriptomics of various cultivars can provide useful information for a better understanding of the physiological and molecular processes involved in mild salinity tolerance in Australian bread wheat cultivars. The high-throughput phenotyping system at The Plant Accelerator of the Australian Plant Phenomics Facility has permitted a systematic analysis of the transcriptome profiles of bread wheat plants under well-controlled conditions and shed light on mild salinity-tolerance mechanisms represented by gene expression in leaf sheath during the early “osmotic” phase of salinity response and adaptation.

## Supporting Information

S1 Table Primer sets for quantitative RT-PCR analysis. (XLSX)

S2 Table Differentially downregulated genes during salinity stress in Drysdale and Krichauff when compared to Berkut and Gladius. Listed are genes whose expression was reduced more than five-fold in Drysdale and Krichauff compared with the other cultivars under salinity stress conditions. *p* values of <0.01 were included. Asterisk indicates stress- and/or abscisic acid treatment-inducible gene in rice and *Arabidopsis*. ^a^Probe name as given by Agilent 44K wheat gene expression array (Design ID: 022297). ^b^Locus indicates GenBank accession. ^c^Load ID as given by RAP-DB. ^d^AGI, Arabidopsis Genome Initiative. ^e^Description as given by The Institute for Genomic Research database.
(XLSX)

S3 Table Differentially downregulated genes during biomass accumulation under control conditions in Berkut. Listed are genes whose expression was reduced more than five-fold in Berkut compared with the other cultivars under control conditions. *p* values of <0.01 were included. Asterisk indicates stress- and/or abscisic acid treatment-inducible gene in rice and *Arabidopsis*. ^a^Probe name as given by Agilent 44K wheat gene expression array (Design ID: 022297). ^b^Locus indicates GenBank accession. ^c^Load ID as given by RAP-DB. ^d^AGI, Arabidopsis Genome Initiative. ^e^Description as given by The Institute for Genomic Research database.
(XLSX)

S4 Table Differentially downregulated genes during salinity stress in Krichauff. Listed are genes whose expression was reduced more than five-fold in Krichauff compared with the other cultivars under salinity stress conditions. *p* values of <0.01 were included. Asterisk indicates stress- and/or abscisic acid treatment-inducible gene in rice and *Arabidopsis*. ^a^Probe name as given by Agilent 44K wheat gene expression array (Design ID: 022297). ^b^Locus indicates GenBank accession. ^c^Load ID as given by RAP-DB. ^d^AGI, Arabidopsis Genome Initiative. ^e^Description as given by The Institute for Genomic Research database.
(XLSX)

S5 Table Differentially downregulated genes during salinity stress in Drysdale. Listed are genes whose expression was reduced more than five-fold in Drysdale compared with the other cultivars under salinity stress conditions. *p* values of <0.01 were included. Asterisk indicates stress- and/or abscisic acid treatment-inducible gene in rice and *Arabidopsis*. ^a^Probe name as given by Agilent 44K wheat gene expression array (Design ID: 022297). ^b^Locus indicates GenBank accession. ^c^Load ID as given by RAP-DB. ^d^AGI, Arabidopsis Genome Initiative. ^e^Description as given by The Institute for Genomic Research database.
(XLSX)

S1 Fig Experimental layout with wheat varieties referred to the first experiment. (TIF)

S2 Fig Experimental layout with wheat varieties referred to the second experiment. (TIF)

S3 Fig Sequential monitoring of shoot biomass under conditions of salinity stress. At the emergence of leaf 4, seedlings of (A) Berkut (blue diamonds, SE, n = 8), (B) Krichauff (orange triangles, SE, n = 8), (C) Gladius (green squares, SE, n = 8) or (D) Drysdale (red circles, SE, n = 7) cultivars grown with no added NaCl (open) or treated with 75 mM NaCl (filled), and digital images captured with an RGB camera. (E) Exponential curves were fitted to the data and relative growth rate (RGR) calculated for the four cultivars.
(TIF)

S4 Fig Clustering analysis of variation in gene expression under salinity stress for four cultivars. (A) Hierarchical clustering of genes up- or downregulated with or without salinity stress in the four cultivars. Microarray analysis was performed at three time points (1, 2 or 3 days after treatment), and gene expression profiles with a five-fold or greater difference in expression were integrated for the treatment period. The condition tree of hierarchical classifications was performed with k-means of clustering algorithms. The Euclidean distances were calculated with the Manhattan method as the distance metric. (B) 3D PCA for the four cultivars. The gene expression clusters of Berkut (blue diamonds), Krichauff (orange triangles), Gladius (green squares) or Drysdale (red circles) under control conditions (open) and under salinity stress (filled) were shown. The Tracy-Widom (TM) test was used to perform the principal component analysis with the GeneSpring GX software.
(TIF)

S5 Fig Confirmation of the microarray data by qRT-PCR analysis. The scatter plot shows the relative gene expression ratio of log_2_ (the signal intensity under saline conditions / the signal intensity under control conditions) between qRT-PCR analysis (*X* axis) and microarray analysis (*Y* axis). The six types of figures indicate each wheat gene among four cultivars. The blue, orange, green, and red colors indicate the data from Berkut, Krichauff, Gladius, and Drysdale, respectively. Correlation coefficient (*R* ^2^) was 0.8418.
(TIF)
