# Supplementary figures and images for: Comparison of Leaf Sheath Transcriptome Profiles with Physiological Traits of Bread Wheat Cultivars under Salinity Stress
Source: PLoS One. 2015 Aug 5;10(8):e0133322. doi: 10.1371/journal.pone.0133322 (PMC4526543; doi:10.1371/journal.pone.0133322)

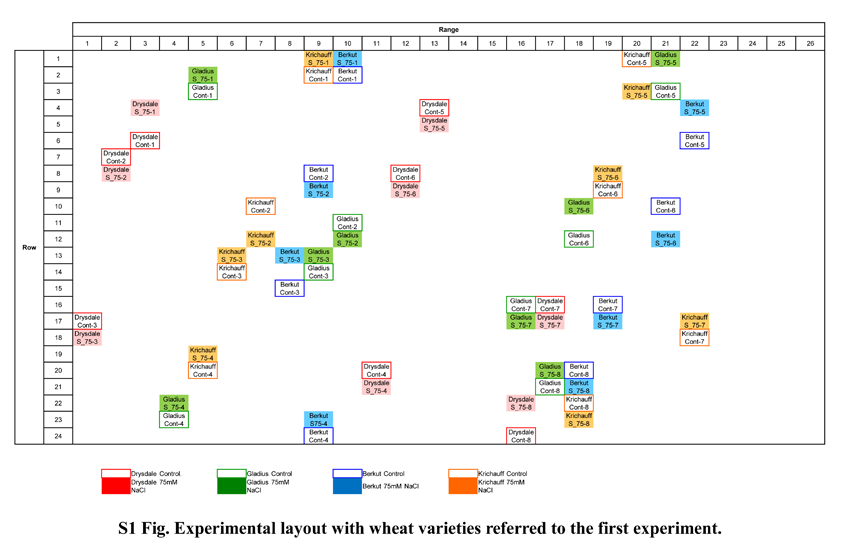

Supplement: S1 Fig — (TIF) [file pone.0133322.s006.tif]

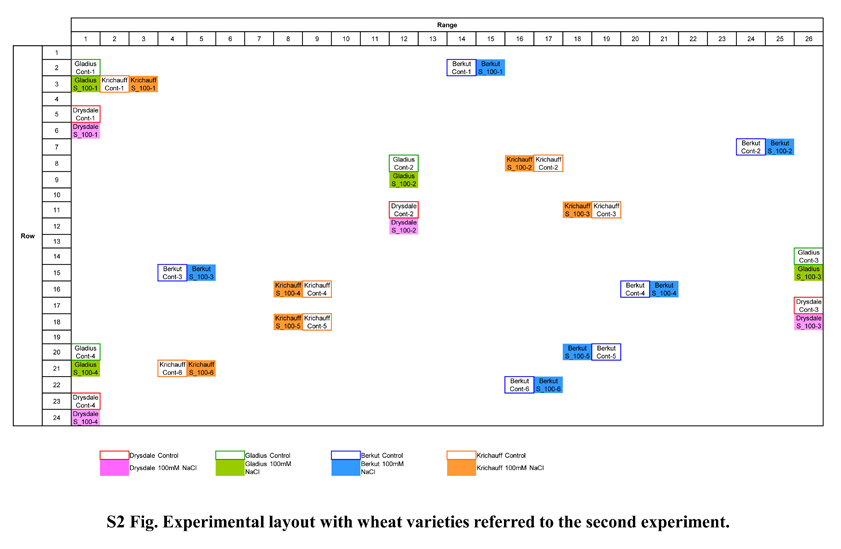

Supplement: S2 Fig — (TIF) [file pone.0133322.s007.tif]

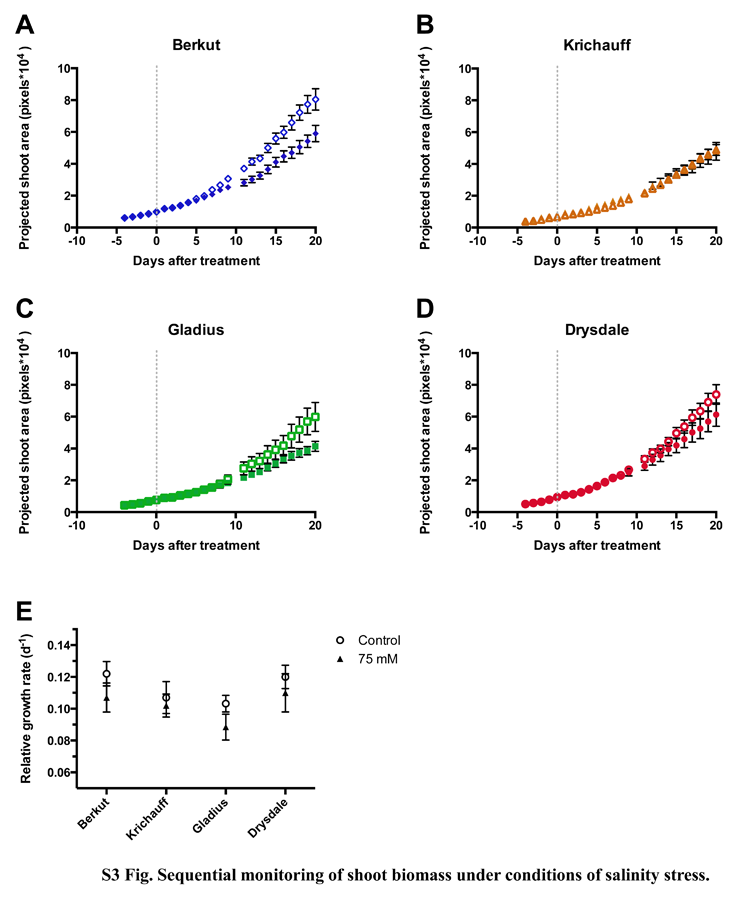

Supplement: S3 Fig — At the emergence of leaf 4, seedlings of (A) Berkut (blue diamonds, SE, n = 8), (B) Krichauff (orange triangles, SE, n = 8), (C) Gladius (green squares, SE, n = 8) or (D) Drysdale (red circles, SE, n = 7) cultivars grown with no added NaCl (open) or treated with 75 mM NaCl (filled), and digital images captured with an RGB camera. (E) Exponential curves were fitted to the data and relative growth rate (RGR) calculated for the four cultivars. (TIF) [file pone.0133322.s008.tif]

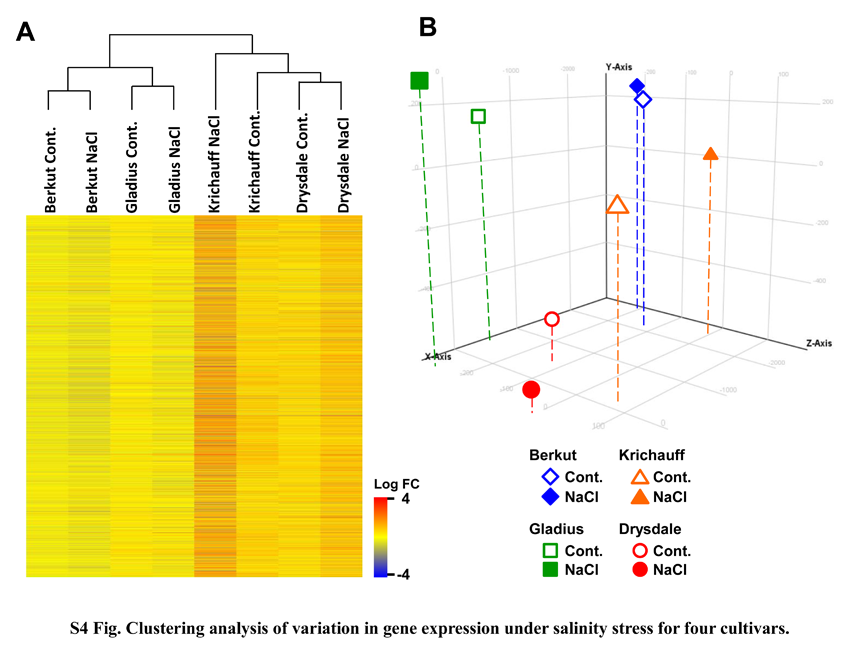

Supplement: S4 Fig — (A) Hierarchical clustering of genes up- or downregulated with or without salinity stress in the four cultivars. Microarray analysis was performed at three time points (1, 2 or 3 days after treatment), and gene expression profiles with a five-fold or greater difference in expression were integrated for the treatment period. The condition tree of hierarchical classifications was performed with k-means of clustering algorithms. The Euclidean distances were calculated with the Manhattan method as the distance metric. (B) 3D PCA for the four cultivars. The gene expression clusters of Berkut (blue diamonds), Krichauff (orange triangles), Gladius (green squares) or Drysdale (red circles) under control conditions (open) and under salinity stress (filled) were shown. The Tracy-Widom (TM) test was used to perform the principal component analysis with the GeneSpring GX software. (TIF) [file pone.0133322.s009.tif]

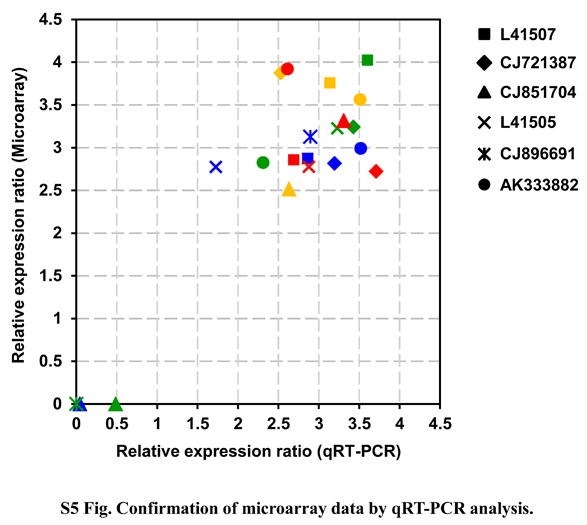

Supplement: S5 Fig — The scatter plot shows the relative gene expression ratio of log2 (the signal intensity under saline conditions / the signal intensity under control conditions) between qRT-PCR analysis (X axis) and microarray analysis (Y axis). The six types of figures indicate each wheat gene among four cultivars. The blue, orange, green, and red colors indicate the data from Berkut, Krichauff, Gladius, and Drysdale, respectively. Correlation coefficient (R 2) was 0.8418. (TIF) [file pone.0133322.s010.tif]
